# *Leuconostoc Mesenteroides* Growth in Food Products: Prediction and Sensitivity Analysis by Adaptive-Network-Based Fuzzy Inference Systems

**DOI:** 10.1371/journal.pone.0064995

**Published:** 2013-05-21

**Authors:** Hue-Yu Wang, Ching-Feng Wen, Yu-Hsien Chiu, I-Nong Lee, Hao-Yun Kao, I-Chen Lee, Wen-Hsien Ho

**Affiliations:** 1 Department of Pharmacy, Chi Mei Medical Center, Tainan, Taiwan; 2 Center for Fundamental Science, Kaohsiung Medical University, Kaohsiung, Taiwan; 3 Department of Healthcare Administration and Medical Informatics, Kaohsiung Medical University, Kaohsiung, Taiwan; University of Ulm, Germany

## Abstract

**Background:**

An adaptive-network-based fuzzy inference system (ANFIS) was compared with an artificial neural network (ANN) in terms of accuracy in predicting the combined effects of temperature (10.5 to 24.5°C), pH level (5.5 to 7.5), sodium chloride level (0.25% to 6.25%) and sodium nitrite level (0 to 200 ppm) on the growth rate of *Leuconostoc mesenteroides* under aerobic and anaerobic conditions.

**Methods:**

The ANFIS and ANN models were compared in terms of six statistical indices calculated by comparing their prediction results with actual data: mean absolute percentage error (MAPE), root mean square error (RMSE), standard error of prediction percentage (SEP), bias factor (B_f_), accuracy factor (A_f_), and absolute fraction of variance (*R* ^2^). Graphical plots were also used for model comparison.

**Conclusions:**

The learning-based systems obtained encouraging prediction results. Sensitivity analyses of the four environmental factors showed that temperature and, to a lesser extent, NaCl had the most influence on accuracy in predicting the growth rate of *Leuconostoc mesenteroides* under aerobic and anaerobic conditions. The observed effectiveness of ANFIS for modeling microbial kinetic parameters confirms its potential use as a supplemental tool in predictive mycology. Comparisons between growth rates predicted by ANFIS and actual experimental data also confirmed the high accuracy of the Gaussian membership function in ANFIS. Comparisons of the six statistical indices under both aerobic and anaerobic conditions also showed that the ANFIS model was better than all ANN models in predicting the four kinetic parameters. Therefore, the ANFIS model is a valuable tool for quickly predicting the growth rate of *Leuconostoc mesenteroides* under aerobic and anaerobic conditions.

## Introduction

Growth prediction models are now widely used informative tools for rapid and cost-effective assessment of microbial growth for product development, risk assessment, and education purposes [1]. In recent studies of shelf life in food products, microbiologists have used predictive models to forecast spoilage caused by the growth of micro-organisms. Despite the major technological advances in the food industry in recent years, fungal spoilage of food commodities remains a major cause of economic losses for food producers and an important health concern for regulatory agencies. Therefore, improved understanding of fungal growth in foods, particularly those factors associated with new manufacturing processing and packaging techniques, is urgently needed [2]. Fungi degrade the organoleptic properties of foods by producing visible mycelium, and fungal contamination is often implicated in off-flavor food products. In addition to the economic effects of consumer rejection, the diminished nutritional value and, more importantly, the production of potentially carcinogenic toxic metabolites, pose a public health risk [3]. Improvements in food quality and safety require the development of appropriate fungal growth prediction tools. For many years, research in predictive microbiology has focused on food-borne pathogens whereas models for predicting growth in filamentous fungi have received relatively less attention [4]. Recently, however, the situation has changed, and the literature now shows a growing number of studies of models for this purpose [5]–[7].

*Leuconostoc mesenteroides* (*LM*) is a common spoilage microorganism in cooked meat products. These bacteria can alter food products by fermentation of sugars, which forms lactic acid. By contributing to slime mold formation and CO_2_ production, they also degrade the smells and flavors of food products. Because the resulting sensorial qualities of the product can make it unacceptable for consumption [8], spoilage caused by these microorganisms is potentially a major cause of economic loss in the food industry. Therefore, a tool is needed for predicting the growth capacity of this microorganism to multiply in a food product under conditions that typically occur while processing, preserving, storing and distributing foods. An effective tool for predicting shelf life would help to reduce economic losses from deterioration of food. In an earlier study of tools for predicting *LM* growth rates under aerobic and anaerobic conditions, Zurera-Cosano et al. [9] used response surface methodology (RSM) to compare the combined effects of different temperatures, pH levels, sodium chloride levels and sodium nitrite levels on the accuracy of *LM* growth rate predictions under aerobic and anaerobic conditions. The RSM models showed potential use for estimating shelf life in food products. However, a subsequent study by Garcia-Gimeno et al. [10] showed that an artificial neural network (ANN) model was more accurate than RSM for predicting *LM* growth given similar environmental conditions.

A literature review shows that most studies in this line of research have used ANN models for predicting the growth of spoilage microorganisms in food products [7] because ANNs can handle high-level nonlinearities, numerous parameters and missing information [11]–[15]. Hajmeer et al. [16] developed an ANN model of *Shigella flexneri* growth and reported that it outperformed regression equations in terms of mean absolute percentage error (MAPE) and coefficient of determination. Again, however, a noted limitation of the ANN model was its high complexity. Geeraerd et al. [17] reported that an ANN model was superior to conventional microbiological models in terms of accuracy in predicting the effects of temperature, pH and NaCl on microbial activity. Jeyamkondan et al. [18] reported that, for predicting growth in *Aeromonas hydrophila*, *S. flexneri* and *Brochothrix thermosphacta*, ANN models were superior to statistical models in terms of root mean square error (RMSE), mean relative percentage error (MRPE) and MAPE. Lou and Nakai [19] applied response surface methodology, a Cerf model and an ANN model in a study of the effects of temperature, water activity and pH on the thermal inactivation of *Listeria monocytogenes*. Again, the network-based approach proved superior in terms of RMSE and coefficient of determination. Lou and Nakai [20] further showed that ANN was more accurate than response surface methodology in a study of the effects of temperature, water activity and dissolved CO_2_ concentration on the kinetic parameters of *Lactobacillus sake*. In Garcia-Gimeno et al. [21], another comparison between ANNs and RSM models for predicting growth rates in *L. plantarum* and *E. coli* showed that, although the ANN models had higher complexity, they had lower standard error of prediction percentage (SEP) terms compared to the statistical models. Panagou and Kodogiannis [7] compared ANN methods and polynomial methods of modeling the joint effects of water activity, pH level and temperature to predict the maximum growth rate of ascomycetous fungus *Monascus ruber.* Comparisons of six statistical indices, *i.e.*, coefficient of determination, RMSE, MRPE, MAPE, SEP, bias factor (B_f_), and accuracy factor (A_f_), confirmed that, for modeling microbial kinetic parameters, ANNs are an acceptable alternative to polynomial methods.

For describing relationships between different combinations of inputs and outputs such as those that must be determined for accurately predicting growth in spoilage microorganisms, ANN is currently the most widely used technique. A recent literature review shows that the use of adaptive-network-based fuzzy inference system (ANFIS) [22], [23] for such purposes is relatively rare. Jang [22] implemented ANFIS in an adaptive fuzzy neural network framework by using a hybrid learning procedure combining back-propagation gradient descent and least square methods. Since the membership functions in the resulting fuzzy inference system were iteratively adjustable according to a given training set of inputs and outputs, the ANFIS could effectively map input–output relationships according to both human knowledge (in the form of fuzzy if–then rules) and stipulated input–output data pairs [24], [25]. In the current study, ANFIS was used to model the relationship between predicted and actual *LM* growth rates under various conditions. Therefore, this study evaluated the accuracy of ANFIS in predicting *LM* growth rates under aerobic and anaerobic conditions. The ANFIS and ANN models were then compared in terms of their accuracy in predicting *LM* growth under varying experimental conditions, including temperature, pH, salt, nitrite concentrations, and under aerobic and anaerobic conditions. Tables 1 and 2 define the four parameters used to compare the ANFIS and ANN models [10] for predicting *LM* growth under aerobic and anaerobic conditions. The ANFIS model was trained using Gaussian membership functions. Some experimental results obtained by ANFIS method were also compared with those obtained by ANN methods in an earlier study by Garcia-Gimeno et al. [10]. Finally, sensitivity analyses were performed to identify the environmental factors that had the largest effects on the accuracy of the predictions of *LM* growth rate under aerobic and anaerobic conditions.

**Table 1:** Average values for observed (OBS) growth rate (G_r_, h^−1^) and average values estimated by ANN [10] and by ANFIS models of *Leuconostoc mesenteroides* growth under aerobic conditions during model development with the training data set [10].

T(°C)	pH	NaCl(%)	NaNO_2_ (ppm)	G_r_ (h^−1^)		
				OBS	ANN	ANFIS
10.5	6.5	3.25	100	0.141	0.147	0.141
14.0	6.0	1.75	50	0.178	0.175	0.178
14.0	6.0	1.75	150	0.160	0.167	0.160
14.0	6.0	4.75	50	0.147	0.143	0.147
14.0	6.0	4.75	150	0.138	0.143	0.138
14.0	7.0	1.75	50	0.200	0.200	0.200
14.0	7.0	1.75	150	0.183	0.188	0.183
14.0	7.0	4.75	50	0.153	0.145	0.153
14.0	7.0	4.75	150	0.146	0.144	0.146
17.5	5.5	3.25	100	0.114	0.117	0.114
17.5	7.5	3.25	100	0.180	0.180	0.180
17.5	6.5	3.25	0	0.194	0.187	0.191
17.5	6.5	3.25	200	0.165	0.167	0.165
17.5^a^	6.5	3.25	100	0.177	0.176	0.176
17.5^a^	6.5	3.25	100	0.178	0.176	0.176
17.5^a^	6.5	3.25	100	0.176	0.176	0.176
17.5^a^	6.5	3.25	100	0.176	0.176	0.176
17.5^a^	6.5	3.25	100	0.176	0.176	0.176
17.5^a^	6.5	3.25	100	0.177	0.176	0.176
17.5	6.5	6.25	100	0.152	0.171	0.152
17.5	6.5	0.25	100	0.369	0.370	0.370
21.0	6.0	1.75	50	0.347	0.348	0.347
21.0	6.0	1.75	150	0.317	0.318	0.317
21.0	6.0	4.75	50	0.324	0.317	0.324
21.0	6.0	4.75	150	0.294	0.194	0.294
21.0	7.0	1.75	50	0.380	0.387	0.380
21.0	7.0	1.75	150	0.362	0.352	0.362
21.0	7.0	4.75	50	0.328	0.332	0.328
21.0	7.0	4.75	150	0.308	0.309	0.308
24.5	6.5	3.25	100	0.422	0.424	0.416

a Center point conditions.

**Table 2:** Average values for observed (OBS) growth rate (G_r_, h^−1^) and average values estimated by ANN [10] and by ANFIS models of *Leuconostoc mesenteroides* growth under anaerobic conditions during model development with the training data set [10].

T(°C)	pH	NaCl(%)	NaNO_2_ (ppm)	G_r_ (h^−1^)		
				OBS	ANN	ANFIS
10.5	6.5	3.25	100	0.106	0.129	0.106
14.0	6.0	1.75	50	0.161	0.155	0.161
14.0	6.0	1.75	150	0.149	0.148	0.149
14.0	6.0	4.75	50	0.139	0.128	0.139
14.0	6.0	4.75	150	0.120	0.119	0.120
14.0	7.0	1.75	50	0.180	0.174	0.180
14.0	7.0	1.75	150	0.168	0.170	0.168
14.0	7.0	4.75	50	0.142	0.130	0.142
14.0	7.0	4.75	150	0.130	0.128	0.130
17.5	5.5	3.25	100	0.103	0.101	0.103
17.5	7.5	3.25	100	0.169	0.173	0.169
17.5	6.5	3.25	0	0.191	0.187	0.188
17.5	6.5	3.25	200	0.157	0.158	0.157
17.5^a^	6.5	3.25	100	0.172	0.171	0.172
17.5^a^	6.5	3.25	100	0.172	0.171	0.172
17.5^a^	6.5	3.25	100	0.170	0.171	0.172
17.5^a^	6.5	3.25	100	0.176	0.171	0.172
17.5^a^	6.5	3.25	100	0.178	0.171	0.172
17.5^a^	6.5	3.25	100	0.167	0.171	0.172
17.5	6.5	6.25	100	0.141	0.163	0.141
17.5	6.5	0.25	100	0.363	0.364	0.363
21.0	6.0	1.75	50	0.336	0.346	0.336
21.0	6.0	1.75	150	0.312	0.309	0.312
21.0	6.0	4.75	50	0.323	0.311	0.323
21.0	6.0	4.75	150	0.269	0.267	0.269
21.0	7.0	1.75	50	0.363	0.363	0.363
21.0	7.0	1.75	150	0.337	0.335	0.337
21.0	7.0	4.75	50	0.313	0.313	0.313
21.0	7.0	4.75	150	0.296	0.287	0.296
24.5	6.5	3.25	100	0.409	0.410	0.408

a Center point conditions.

## Materials and Methods

### ANFIS architecture

The ANFIS multilayer feed-forward network of nodes and directional links combines the learning capabilities of an ANN with the reasoning capabilities of fuzzy logic. The ANNs and fuzzy inference systems (FISs) are complementary technologies in the design of adaptive intelligent systems. The ANNs, which learn from scratch by adjusting the interconnections among neurons, are noted for their generalization capability. That is, a properly trained ANN can correctly match a set of new input data to output data. The FIS is a popular computing framework based on fuzzy set theory, fuzzy if–then rules, and fuzzy reasoning. For a given set of if-then rules, an FIS can perform nonlinear mapping from its input space to its output space [22]–[25].

By storing its essential components in a knowledge base, fuzzy reasoning enables an FIS to infer an overall output value according to human expertise. However, no systematic method of transforming the experience and knowledge of human experts to an FIS knowledge base has been developed. To build an FIS, the fuzzy sets, fuzzy operators and knowledge base must be specified. To construct an ANN for an application, the architecture and learning algorithm must be specified. The homogenous structure of an ANN complicates the extraction of structured knowledge from the weights of interconnections between its neurons. The a priori knowledge of human experts is often needed to solve practical problems. However, encoding prior knowledge into an ANN is difficult. The ANFIS combines the advantage of FIS, *i.e.*, its learning capability, with the advantage of ANN, *i.e.*, its formation of a linguistic rule base. To obtain an ANFIS that can share data structures and knowledge representations, ANN and FIS are typically integrated by using a special ANN architecture to represent FIS [22]–[25].

**Figure 1:**
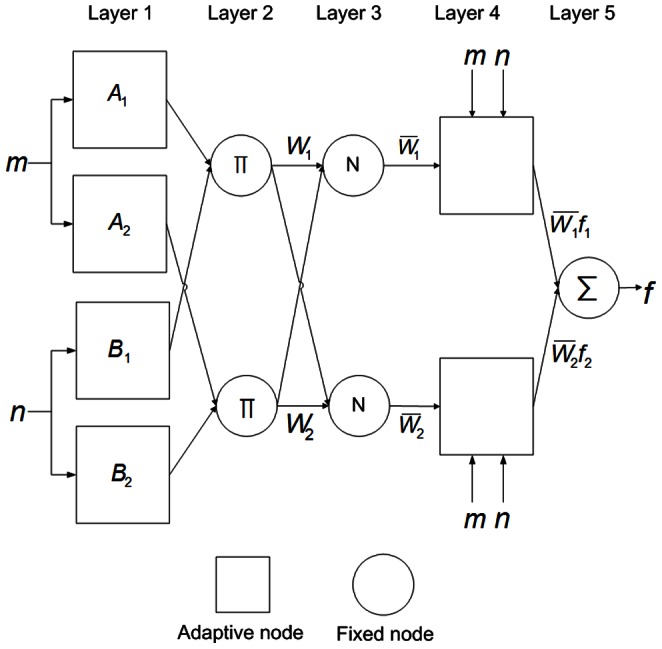
Framework of adaptive network-based fuzzy inference system. Layer 1: fuzzification layer; Layer 2: production layer; Layer 3: normalization layer; Layer 4: de-fuzzification layer; Layer 5: total output layer.

The hybrid network structure of ANFIS can achieve prediction capabilities superior to those of ANN alone and fuzzy logic techniques alone. By analyzing mapping relationships between input and output data, ANFIS optimizes the distribution of membership functions by using a back-propagation gradient descent algorithm either alone or combined with a least-squares method [22]. The ANFIS uses fuzzy if–then rules involving premise and consequent parts of a Sugeno-type fuzzy inference system [22]. Figure 1 shows how the description of this system can be simplified as an inference system of inputs *m* and *n* and output *f* [22]. The corresponding ANFIS architecture is also shown. The five-layer system ANFIS architecture includes a fuzzification layer (Layer 1), a production layer (Layer 2), a normalization layer (Layer 3), a de-fuzzification layer (Layer 4), and a total output layer (Layer 5) [22].

In the first layer, which is the fuzzification layer, *m* and *n* are the inputs of nodes 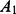 and 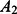 and nodes 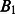 and 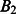, respectively. Each node *i* in this layer is a square node (adaptive node 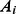 and 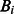, 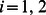). The linguistic labels used in fuzzy theory to divide membership functions are 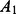 (small), 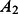 (large), 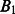 (small) and 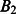 (large). The Gaussian membership relationship between the output and input functions of this layer can be expressed as
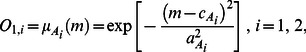
(1a)and
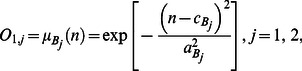
(1b)where 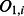 and 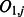 denote the output functions, 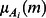 and 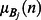 denote the Gaussian membership functions; parameters 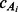 and 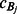 (the center) and parameters 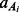 and 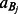 are represented by 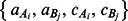 (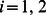 and 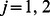,) and denote the nonlinear parameters of the premise part, which are the width of the Gaussian membership function of the *i*th implication of input variables *m* and *n*, respectively.

The second layer, which is the production layer, consists of two circular nodes labeled 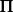, which multiply the incoming signals. The resulting product is used to represent the firing strength of a rule. In this layer, the product operator that performs a generalized AND can be used as the node function. Outputs 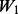 and 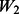 are the weight functions of the next layer. The output of this layer is the product of the input signal, which is defined as follows:
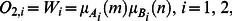
(2)where 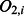 denotes the output of Layer 2.

The third layer is the normalization layer. Each node in this layer is a circular node labeled N. The *i*th node calculates the ratio of the firing strength of the *i*th rule to the sum of the firing strengths of all rules. Its function is to normalize the weight function by applying the following process:
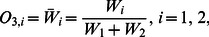
(3)where 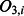 denotes the Layer 3 output.

The fourth layer is the de-fuzzification layer. Each node *i* in this layer is a square node (adaptive node, 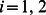). The output equation is 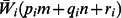, where 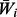 is the output of Layer 3 and where 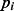, 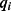, and 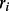 represented by 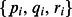 (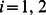) denote the linear parameters or the so-called consequent parameters of the node. The de-fuzzification relationship between the input and output of this layer can be expressed as the output of the *i*th rule as follows:
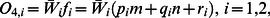
(4)where 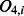 denotes the Layer 4 output.

The fifth layer is the total output layer. The single node in this layer is a circular node labeled 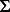, which computes the overall output by summing all incoming signals. The output of this layer is the total of the input signals, which represents the predicted growth rate of *LM*. The results can be written as
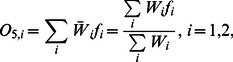
(5)where 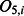 denotes the Layer 5 output.

Figure 1 further shows the typical if-then rules in a first-order Sugeno fuzzy model [24], [25]:
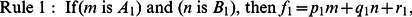
(6a)and
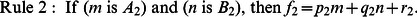
(6b)


**Figure 2:**
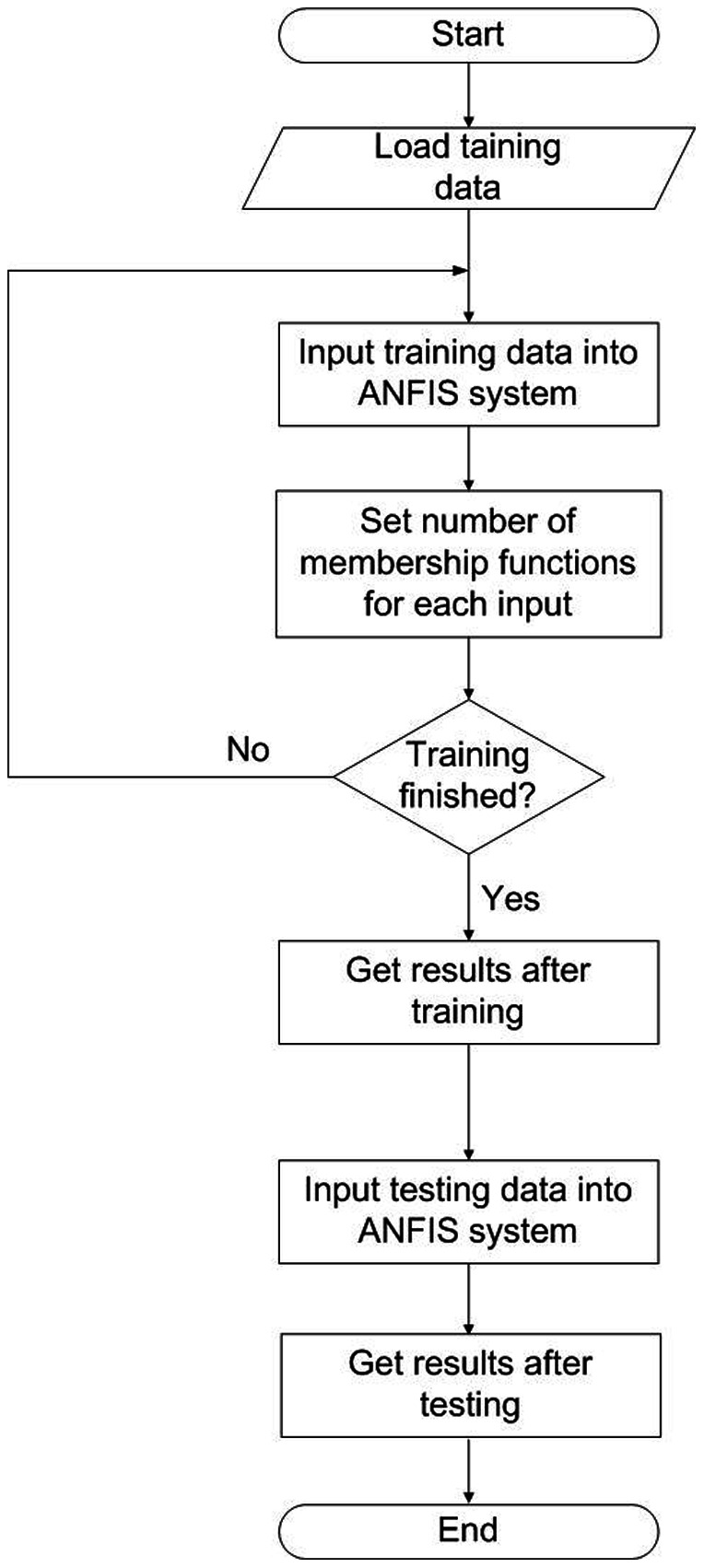
Flowchart of ANFIS for predicting the growth rate of *Leuconostoc mesenteroides*. In the training process, the number of membership functions is set for each input parameter until prediction performance is satisfactory. After the training procedure obtains the training results, the testing data are input into the trained ANFIS model to obtain the testing results.

In the ANFIS architecture, the nonlinear parameters 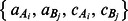
of the premise part and the linear parameters 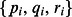 of the consequent part can be trained by using a hybrid learning procedure combining back-propagation gradient descent with least square methods. Figure 2 is a flowchart showing the growth rates predicted by ANFIS. The training data are used to train the nonlinear parameters 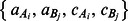
of the premise part and the linear parameters 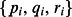 of the consequent part. The number of membership functions is set for each input parameter until prediction performance is satisfactory. After the training results are obtained by this process, the testing data are input into the trained ANFIS model to obtain the testing results. The following sections present the details of the input-output relationships in each ANFIS layer.

Note that the system output is the weighted sum of the results of the rules. The number of fuzzy sets depends on the number of Layer 1 nodes. However, the number of Layer 4 dimensions determines the number of fuzzy rules used in the ANFIS architecture. Therefore, the number of Layer 4 dimensions indicates the complexity and flexibility of the ANFIS architecture. The number of fuzzy rules in an ANFIS is analogous to the number of neurons in an ANN [25].

Like ANNs, an ANFIS network can be trained by supervised learning by using a specified output to provide a target output. The forward pass of the hybrid algorithm of the ANFIS moves the node outputs forward to Layer 4. The consequent linear parameters 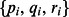 are then obtained by least-squares method [22], [25]. In the backward pass, the error signals propagate backwards, and the premise nonlinear parameters 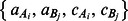 (the centre and the width of the Gaussian membership function) are updated by gradient descent [22], [25]. That is, the premise nonlinear parameters 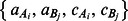 and the consequent linear parameters 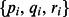 are trained in the ANFIS.

### Evaluation criteria

As described in the literature, the criteria used to compare fitting and prediction accuracy between the ANFIS model and the ANN model were MAPE, RMSE, SEP, B_f_, A_f_, and the absolute fraction of variance (*R* ^2^). The MAPE indicates the relative absolute percentage deviation in experimental values where a lower value implies a better correlation. The equation for calculating MAPE is
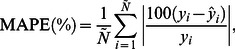
(7)where 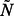 is the number of the points in the data set, 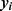 is the observed value, and 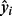 is the prediction value. The RMSE is given by
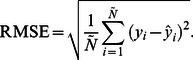
(8)Although unlikely, an RMSE of 0 indicates the best possible fit between predicted and actual values. The SEP(%) is the relative typical deviation in mean prediction values. The advantage of SEP(%) compared to other error measures is that SEP(%) is calculated independently of the magnitude of the measurements. The equation for calculating SEP(%) is
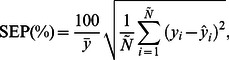
(9)where 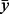 is the mean of observed values. To evaluate the predictive capacity of the proposed model, the following formulas were used to calculate RMSE and SEP(%)together with B_f_ and A_f_ [26] :
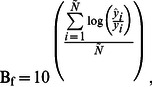
(10)and
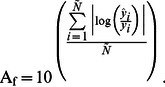
(11)The equation for *R* ^2^ is
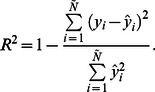
(12)An *R* ^2^ value of 1 indicates a very good fit whereas a value approaching 0 indicates a poor fit.

### Sensitivity analysis of ANFIS output

Sensitivity analysis was performed with ANFIS learning disabled so that network weights would not be affected [27]. The first input varies between its mean plus or minus a user-defined number of standard deviations whereas all other inputs are fixed at their respective means. The ANFIS output is computed and recorded as the percent change above and below the mean channel output. This process is repeated for each input variable [28].

In this so-called ‘sensitivity’ analysis to determine the relative importance of input variables for determining output, 0 indicates a variable that does not affect prediction, and 1.0 indicates a field that completely dominates the prediction [28].

### Data description

Tables 1, 2, 3 and 4 show the Central Composite Design used to obtain temperature (10.5, 14, 17.5, 21 and 24.5°C), pH (5.5, 6, 6.5, 7 and 7.5), NaCl (0.25%, 1.75%, 3.25%, 4.75% and 6.25%) and NaNO_2_ (0, 50, 100, 150, and 200 ppm), respectively, under aerobic and anaerobic conditions [10]. Each of the 25 different factor combinations thus obtained was replicated seven times, and six center point replications were performed to estimate experimental variance. For comparison, Tables 1, 2, 3 and 4 show the growth rate estimations obtained by Garcia-Gimeno et al. [10]. Tables 1 and 2 show the 30 data sets used to train the ANFIS model [10]. Tables 3 and 4 show the 28 testing data sets [10]. The six evaluation criteria are applied to compare fitting and prediction accuracy between the ANFIS model and the ANN model (Table 5 and 6).

**Table 3:** Average values for observed (OBS) growth rate (G_r_, h^−1^) and average values estimated by ANN [10] and by ANFIS models of *Leuconostoc mesenteroides* growth under aerobic conditions using testing data set [10].

T(°C)	pH	NaCl(%)	NaNO_2_ (ppm)	G_r_ (h^−1^)		
				OBS	ANN	ANFIS
10.5	6.5	0.25	50	0.190	0.359	0.189
10.5	6.5	1.75	0	0.182	0.190	0.182
10.5	6.5	1.75	50	0.172	0.184	0.174
10.5	6.5	1.75	100	0.161	0.179	0.161
10.5	6.5	3.25	0	0.162	0.148	0.162
10.5	6.5	3.25	50	0.151	0.148	0.151
10.5	6.5	3.25	100	0.141	0.147	0.141
14.0	7.0	1.75	0	0.230	0.208	0.230
14.0	7.0	4.75	0	0.161	0.146	0.161
17.5	6.5	0.25	50	0.382	0.392	0.382
17.5	6.5	1.75	50	0.350	0.218	0.350
17.5	6.5	1.75	100	0.341	0.208	0.340
17.5	6.5	3.25	50	0.177	0.181	0.184
17.5	6.0	1.75	50	0.290	0.203	0.290
17.5	6.0	3.25	50	0.172	0.175	0.172
17.5	7.0	3.25	50	0.268	0.183	0.268
21.0	6.0	3.25	50	0.339	0.321	0.339
21.0	7.0	3.25	50	0.352	0.339	0.352
21.0	6.0	0.25	0	0.383	0.522	0.383
21.0	6.0	1.75	0	0.372	0.363	0.372
24.5	6.5	3.25	100	0.409	0.424	0.416
24.5	6.5	3.25	50	0.432	0.426	0.432
24.5	6.5	3.25	150	0.394	0.421	0.394
24.5	6.0	1.75	150	0.473	0.408	0.473
24.5	6.0	4.75	50	0.386	0.388	0.386
24.5	6.0	4.75	150	0.308	0.384	0.308
24.5	7.0	4.75	50	0.350	0.422	0.350
24.5	7.0	4.75	150	0.322	0.418	0.322

**Table 4:** Average for observed (OBS) growth rate (G_r_, h^−1^) and average values estimated by ANN [10] and by ANFIS models of *Leuconostoc mesenteroides* growth under anaerobic conditions during model development with the testing data set [10].

T(°C)	pH	NaCl(%)	NaNO_2_ (ppm)	G_r_ (h^−1^)		
				OBS	ANN	ANFIS
10.5	6.5	0.25	50	0.172	0.318	0.172
10.5	6.5	1.75	0	0.161	0.159	0.161
10.5	6.5	1.75	50	0.153	0.158	0.153
10.5	6.5	1.75	100	0.141	0.157	0.141
10.5	6.5	3.25	0	0.128	0.129	0.128
10.5	6.5	3.25	50	0.112	0.129	0.112
10.5	6.5	3.25	100	0.106	0.129	0.106
14.0	7.0	1.75	0	0.214	0.176	0.214
14.0	7.0	4.75	0	0.149	0.131	0.149
17.5	6.5	0.25	50	0.374	0.375	0.374
17.5	6.5	1.75	50	0.305	0.210	0.305
17.5	6.5	1.75	100	0.297	0.202	0.297
17.5	6.5	3.25	50	0.175	0.179	0.181
17.5	6.0	1.75	50	0.274	0.201	0.274
17.5	6.0	3.25	50	0.157	0.176	0.157
17.5	7.0	3.25	50	0.258	0.179	0.258
21.0	6.0	3.25	50	0.332	0.319	0.332
21.0	7.0	3.25	50	0.350	0.322	0.350
21.0	6.0	0.25	0	0.380	0.498	0.380
21.0	6.0	1.75	0	0.369	0.360	0.369
24.5	6.5	3.25	100	0.406	0.410	0.408
24.5	6.5	3.25	50	0.416	0.413	0.416
24.5	6.5	3.25	150	0.389	0.404	0.389
24.5	6.0	1.75	150	0.402	0.406	0.402
24.5	6.0	4.75	50	0.383	0.398	0.383
24.5	6.0	4.75	150	0.334	0.362	0.334
24.5	7.0	4.75	50	0.350	0.408	0.350
24.5	7.0	4.75	150	0.332	0.401	0.332

**Table 5:** Comparison of performance indices between ANN and ANFIS models under aerobic conditions.

Statistical index	Model	Data set	
		Training	Testing
Mean absolute percentage error (%) (MAPE)	ANN	3.06	15.69
	ANFIS	0.18	0.27
Root mean square error (RMSE)	ANN	0.019	0.067
	ANFIS	0.001	0.002
Standard error of prediction percentage (%) (SEP)	ANN	8.45	23.27
	ANFIS	0.58	0.67
Bias factor (B_f_)	ANN	0.99	0.99
	ANFIS	1.00	1.00
Accuracy factor (A_f_)	ANN	1.03	1.17
	ANFIS	1.00	1.00
Absolute fraction of variance (*R* ^2^)	ANN	0.9937	0.9539
	ANFIS	1.0000	1.0000

**Table 6:** Comparison of performance indices between ANN and ANFIS models under anaerobic conditions.

Statistical index	Model	Data set	
		Training	Testing
Mean absolute percentage error (%) (MAPE)	ANN	3.18	14.46
	ANFIS	0.39	0.14
Root mean square error (RMSE)	ANN	0.008	0.053
	ANFIS	0.002	0.001
Standard error of prediction percentage (%) (SEP)	ANN	3.66	19.46
	ANFIS	0.81	0.44
Bias factor (B_f_)	ANN	1.00	1.00
	ANFIS	1.00	1.00
Accuracy factor (A_f_)	ANN	1.03	1.15
	ANFIS	1.00	1.00
Absolute fraction of variance (*R* ^2^)	ANN	0.9989	0.9686
	ANFIS	0.9999	1.0000

To compare the robustness of the ANFIS and ANN models, bootstrap method [29] was used to minimize problems resulting from insufficient data. Five bootstrap data sets derived from the original data set with different numbers of bootstrap samples (30, 60, 90, 120 and 150) were prepared under both aerobic and anaerobic conditions. At this step, the goal was to determine the appropriated number of bootstrap samples needed for model evaluation. Tables 7 and 8 show the means and standard deviations in each variable for five sets of bootstrap data.

**Table 7:** Five bootstrap data sets derived from the original data set under aerobic conditions.

Data sets	T(°C)		pH		NaCl (%)		NaNO_2_ (ppm)		G_r_ (h^−1^)	
	Mean	Standard deviation	Mean	Standard deviation	Mean	Standard deviation	Mean	Standard deviation	Mean	Standard deviation
30	17.4028	0.6159	6.4772	0.0629	3.2917	0.2166	99.7222	6.8381	0.2199	0.0165
60	17.3950	0.5513	6.5000	0.0699	3.2533	0.2215	99.5000	6.5261	0.2218	0.0143
90	17.4144	0.5555	6.5048	0.0785	3.2522	0.2252	99.0370	6.4862	0.2231	0.0142
120	17.4008	0.5507	6.5022	0.0800	3.2342	0.2322	99.4722	6.8769	0.2232	0.0144
150	17.3833	0.5479	6.4982	0.0778	3.2333	0.2282	99.5778	6.8900	0.2228	0.0150

**Table 8:** Five bootstrap data sets derived from original data set under anaerobic conditions.

Data sets	T(°C)		pH		NaCl (%)		NaNO_2_ (ppm)		G_r_ (h^−1^)	
	Mean	Standard deviation	Mean	Standard deviation	Mean	Standard deviation	Mean	Standard deviation	Mean	Standard deviation
30	17.4654	0.4578	6.4922	0.0695	3.2140	0.1937	97.4530	7.6915	0.2153	0.0113
60	17.4767	0.5578	6.5055	0.0624	3.2418	0.2008	99.3013	7.9161	0.2148	0.0149
90	17.4647	0.5417	6.5005	0.0686	3.2583	0.2202	99.3499	7.7815	0.2145	0.0144
120	17.5037	0.5303	6.5000	0.0674	3.2494	0.2073	98.6586	7.7382	0.2153	0.0136
150	17.4742	0.5134	6.4983	0.0684	3.2465	0.2092	98.8759	7.4808	0.2147	0.0129

Bootstrap resampling has been widely used in applied statistics in the past three decades [30] since the bootstrap method was first introduced by Efron in 1979 [29]. Boostrapping is a Monte Carlo-type data augmentation method of resampling with replacement that can be used with observed data. While Monte Carlo techniques usually generate fictitious data, bootstrap resamples in which the originally observed values are replaced can be used to generate multiple bootstrap samples for use as a proxy for the actual independent sample. Each bootstrap sample is a random sub-sample (of a size equal to that of the original sample) in which the actually observed values are replaced. The original sample is considered a “virtual population”, and the sample is repeatedly duplicated. The procedure can be repeated as needed [30].

## Results

First, the ANFIS was trained using 30 data sets (Tables 1 and 2) selected from the 58 data sets obtained in the experiments. After training was completed, another 28 data sets were then used to verify its accuracy in predicting growth rates (Tables 3 and 4). The prediction results are further analyzed and discussed below.

**Figure 3:**
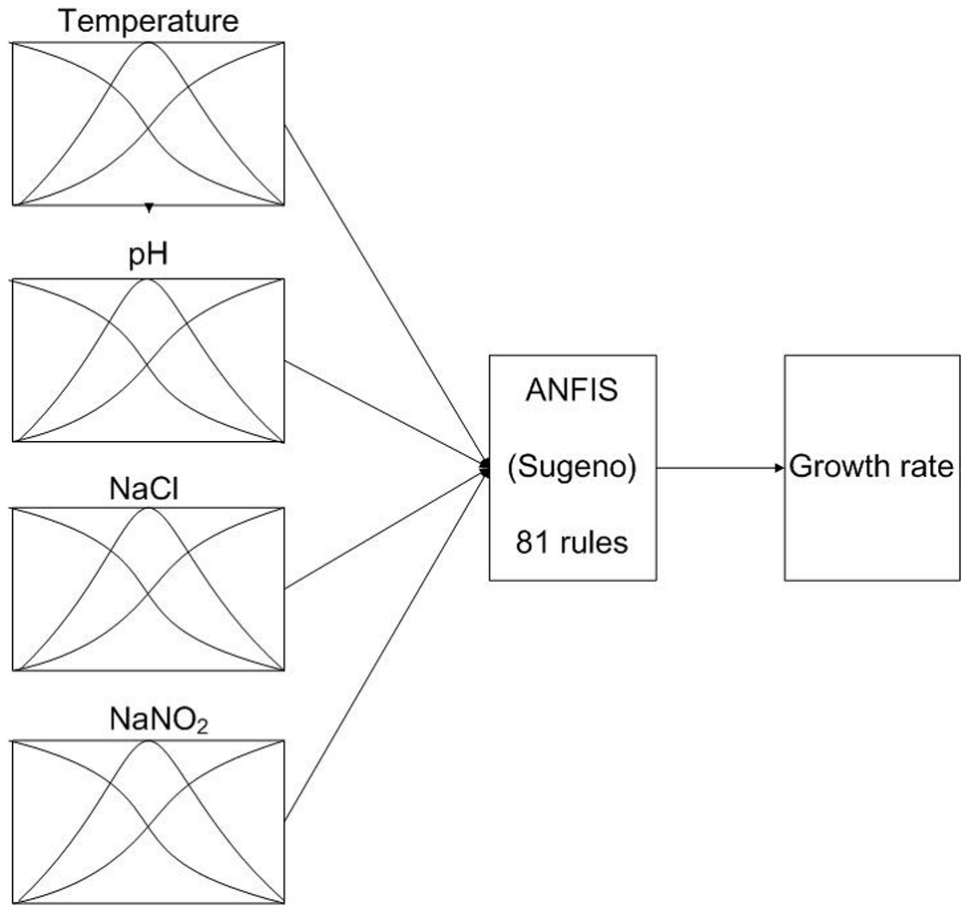
Fuzzy rule architecture of the Gaussian membership function. The four input (temperature, pH, NaCl, NaNO_2_) and one output (growth rate) parameters for the adaptive network-based fuzzy inference system model were used to the predict growth rate of *Leuconostoc mesenteroides* under aerobic and anaerobic conditions. Each input parameter divides three Gaussian membership functions (i.e. small, medium and large areas). The number of fuzzy rules is 81.

**Figure 4:**
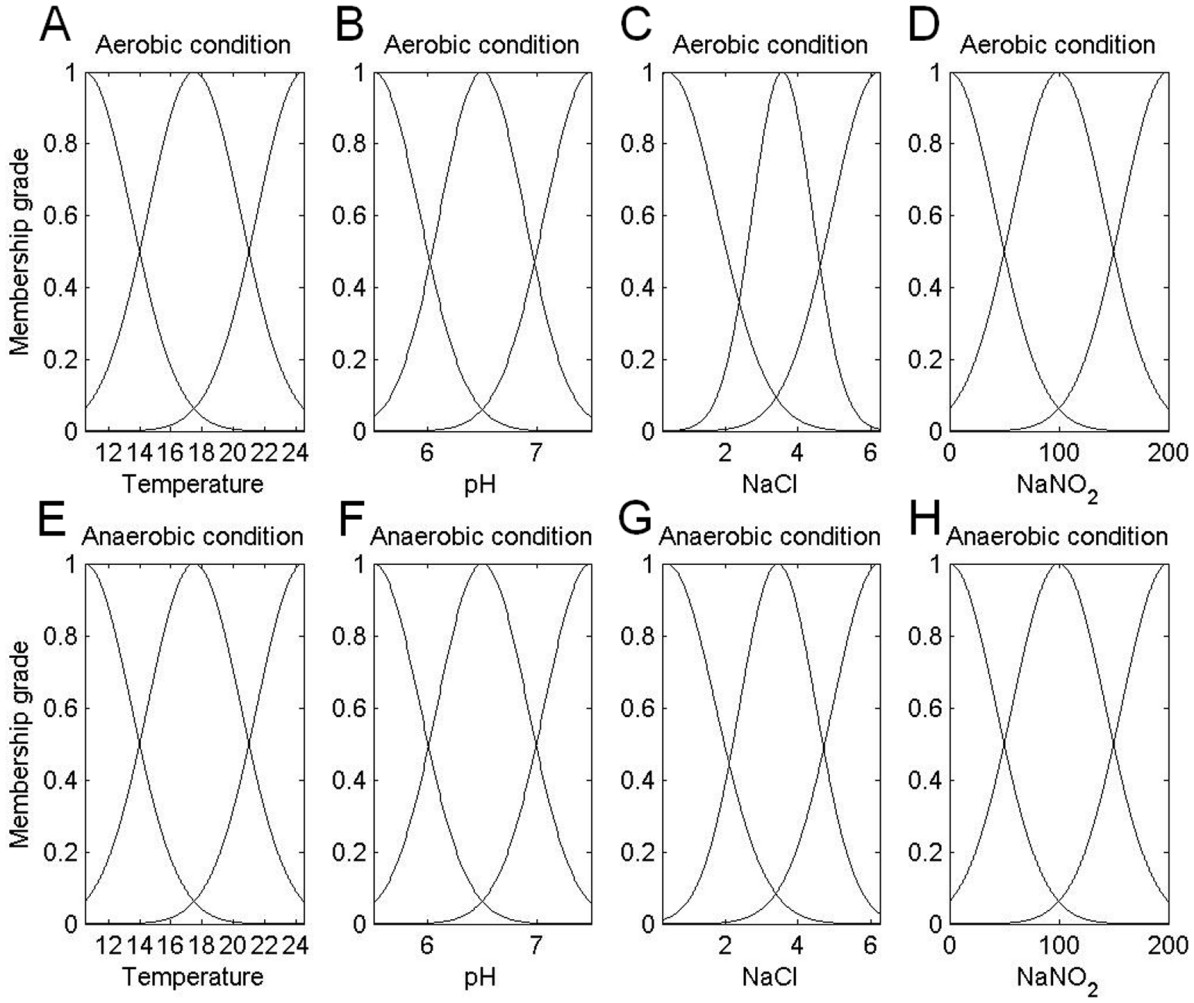
Final Gaussian membership functions of the four input parameters derived by training under aerobic and anaerobic conditions. The adaptive network-based fuzzy inference system was trained using 30 sets of experimental data in 100 learning cycles under aerobic and anaerobic conditions. The final Gaussian membership functions were obtained for the four inputs under aerobic conditions (A) temperature, (B) pH, (C) NaCl and (D) NaNO_2_ and under anaerobic conditions (E) temperature, (F) pH, (G) NaCl and (H) NaNO_2_. Each input divides three Gaussian membership functions (i.e., small, medium and large areas).

Figure 3 shows the fuzzy rule architecture of an ANFIS with Gaussian membership function. The four inputs (temperature, pH, NaCl, and NaNO_2_) and one output (growth rate) of the ANFIS model were designed using MATLAB Fuzzy Logic Toolbox [31]. Each input divides three Gaussian membership functions. Therefore, the architecture in Figure 3 includes 81 fuzzy rules. The ANFIS was trained using 30 sets of experimental data in 100 learning cycles. For each input in the architecture, the Gaussian membership function can be divided into small, medium and large areas. Figure 4 shows the final Gaussian membership functions of the four inputs derived by training under aerobic and anaerobic conditions, respectively.

**Figure 5:**
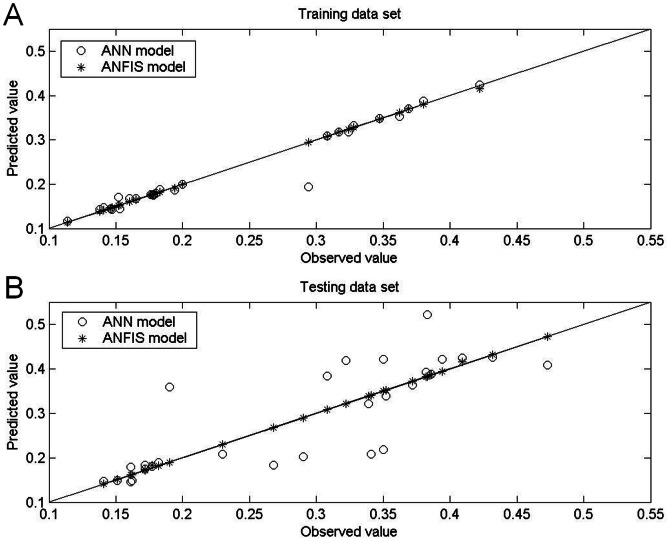
Comparison of actual growth rates for *Leuconostoc mesenteroides* and growth rates predicted by ANFIS model and by ANN model under aerobic conditions. Under aerobic conditions, all specific growth rates predicted by the ANFIS models when using the (A) training data set and the (B) testing data set were closer to the 45° line compared to the rates predicted by the ANN models, which confirmed the superior prediction accuracy of the ANFIS models.

**Figure 6:**
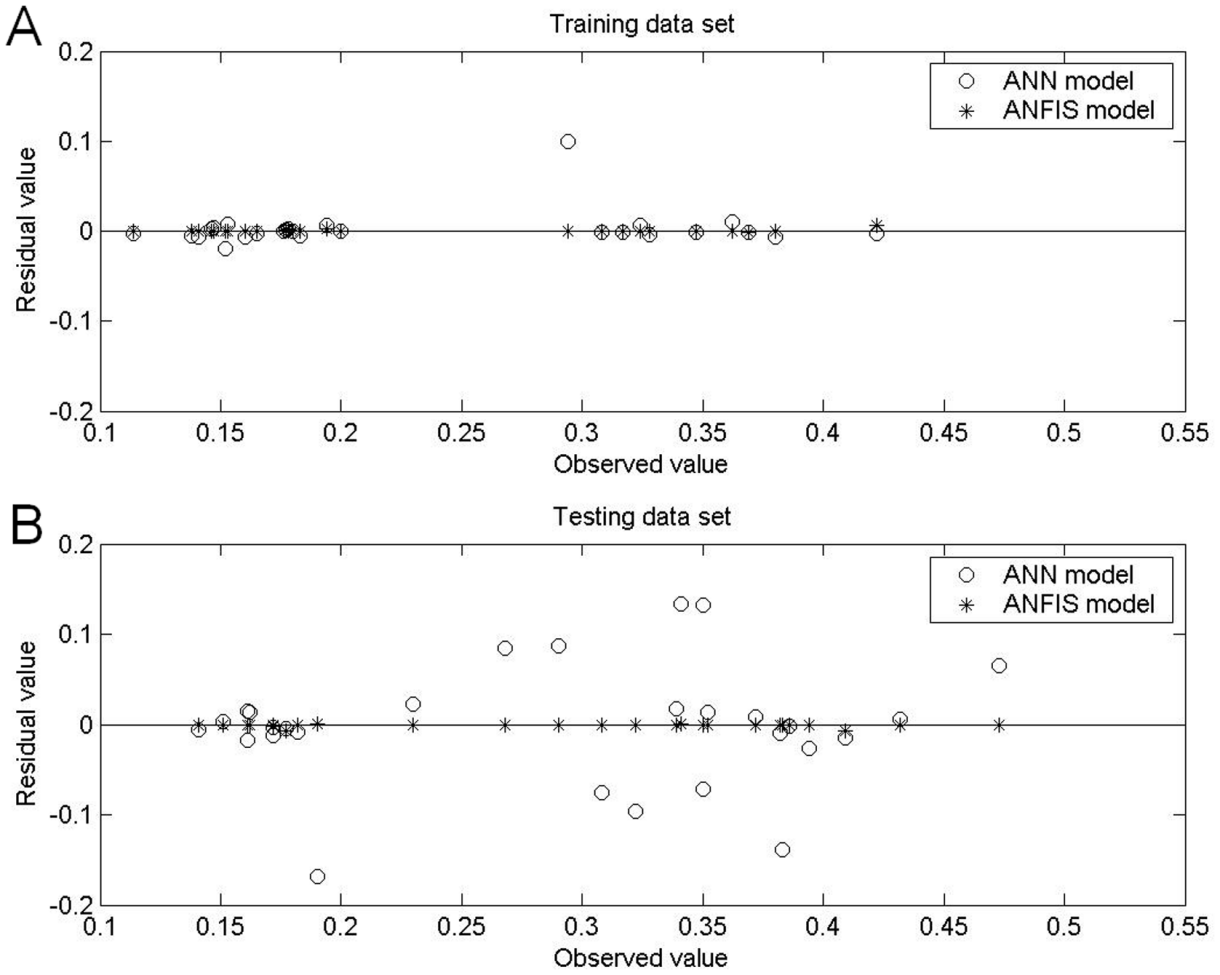
Comparison of residual values (predicted–observed growth rate) of *Leuconostoc mesenteroides* obtained by ANFIS model and by ANN model under aerobic conditions. The spread of residual values was narrower for the ANFIS models for (A) the training data set and for (B) the testing data set, which indicated their better prediction performance under aerobic conditions.

**Figure 7:**
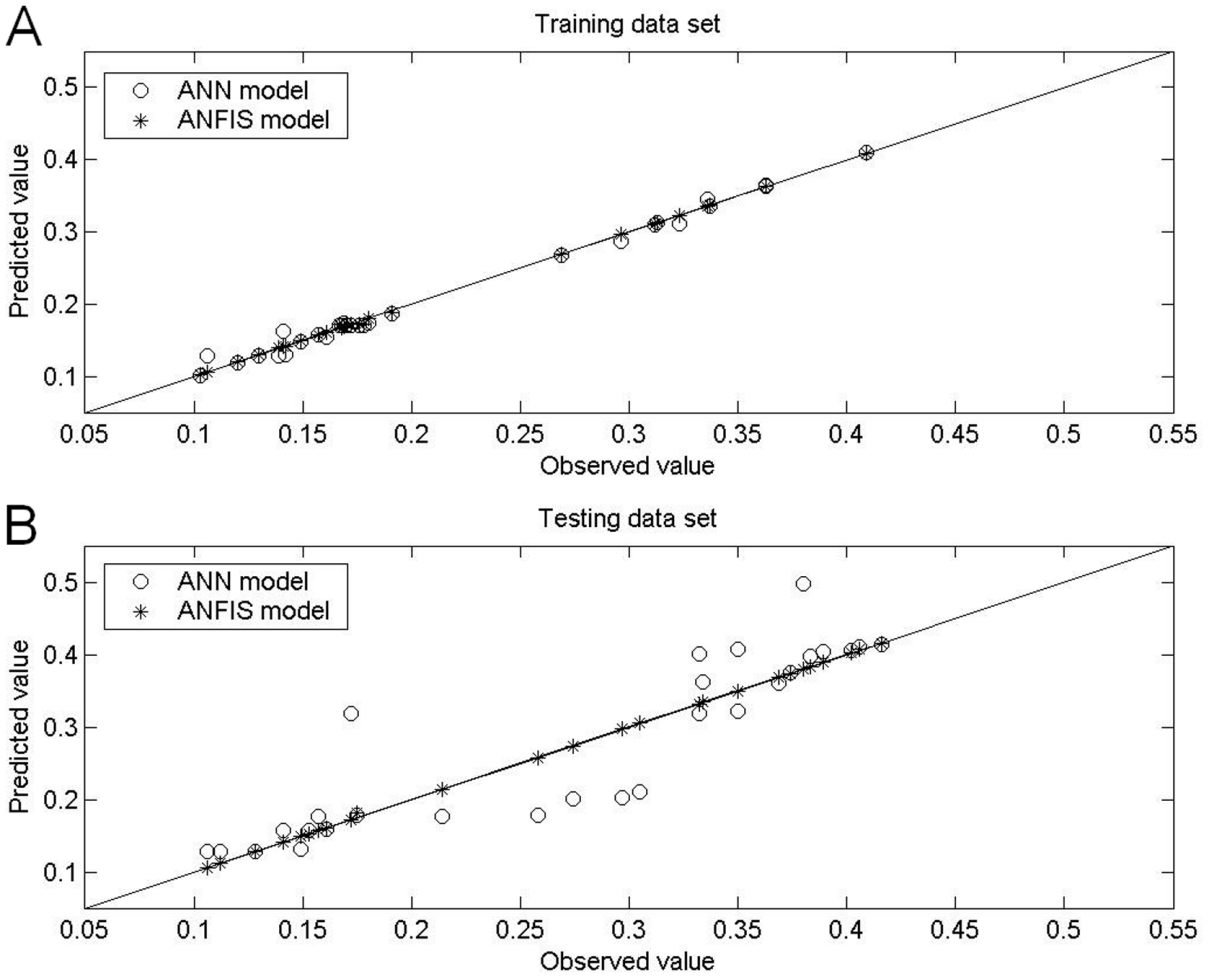
Actual values for growth rate of *Leuconostoc mesenteroides* compared with values predicted by ANFIS model and by ANN model under anaerobic conditions. Under anaerobic conditions, the growth rates predicted by ANFIS models in (A) the training data set and in (B) the testing data set were closer to the 45° line compared to those predicted by the ANN models. In other words, the predictive accuracy of the ANFIS models was higher than that of ANN models under anaerobic conditions.

**Figure 8:**
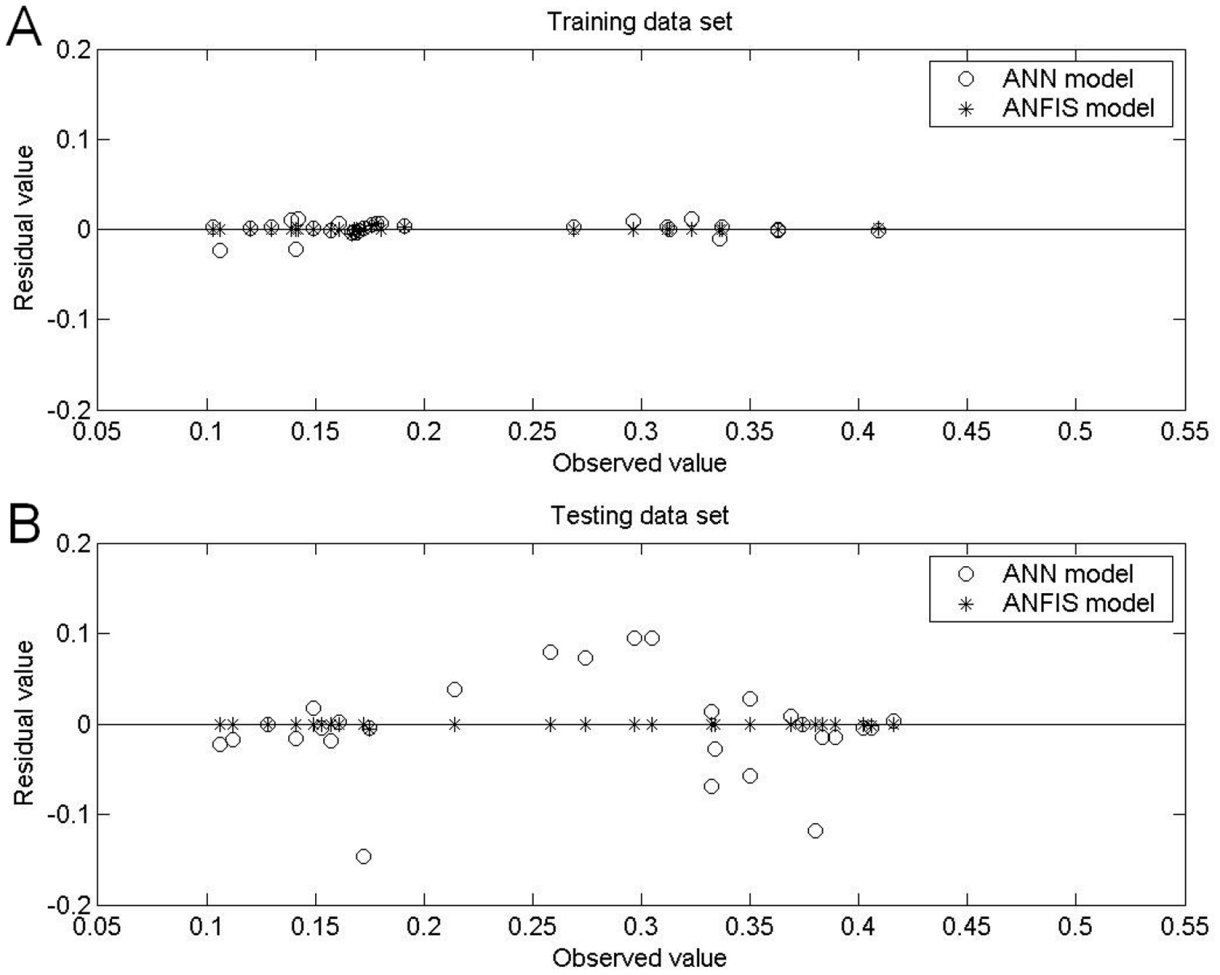
Comparison of residual values (predicted–observed growth rate) of *Leuconostoc mesenteroides* obtained by ANFIS model and by ANN model under anaerobic conditions. The better prediction performance of the ANFIS models under anaerobic conditions was confirmed by their narrower spread of residual values for (A) the training data set and for (B) the testing data set.

Tables 5 and 6 show the prediction results obtained by the ANFIS models under aerobic and anaerobic conditions, respectively, and Figures 5, 6, 7 and 8 show the training and testing data sets. The MAPEs obtained under aerobic conditions are 0.18 for the training data set and 0.27 for the testing data set, and those obtained under aerobic conditions are 0.39 for the training data set and 0.14 for the testing data set. The RMSEs obtained under aerobic conditions are 0.001 for the training data set and 0.002 for the testing data set, and those obtained under anaerobic conditions are 0.002 for the training data set and 0.001 for the testing data set. The SEPs obtained under aerobic conditions are 0.58 for the training data set and 0.67 for the testing data set, and those obtained under anaerobic conditions are 0.81 for the training data set and 0.44 for the testing data set.

Comparisons of performance indices for predictions of the 30 patterns on which the models were trained showed that the ANFIS was more accurate than the ANN. Specifically, the ANFIS models were superior in terms of MAPE, RMSE and SEP (Tables 5 and 6). For the 28 testing data sets, comparisons of model performance again confirmed the superior performance of the ANFIS models, considering the biological variability associated with the experiment. Additionally, measurements of bias and accuracy in the ANFIS models approached unity in all experiments, which indicated good agreement between observations and predictions. The performance difference between the two examined models was also graphically depicted in plots of bias (observed vs. predicted growth rate) and residual bias for all data sets. Generally, the predictions derived by ANFIS models were closer to the line of equity compared to the ANN models (Figures 5 and 7), which indicated the better fit of the ANFIS models. In both the ANFIS and ANN models, residuals were also symmetrically distributed around 0 with no systematic tendency to appear on the positive or negative sides of the graph (Figures 6 and 8). The narrower spread of residual values obtained by the ANFIS models in all data sets also indicated their superior prediction performance.

**Figure 9:**
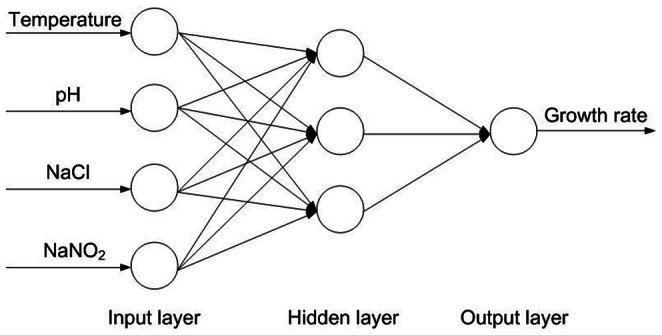
Three-layer feedforward ANN. The input layer includes nodes for four inputs (temperature, pH, NaCl, and NaNO_2_). In the hidden layer, three nodes transfer data to the output layer via a separate weighted logistic transfer function. In the output layer, one node (growth rate) transfers data via a separate weighted identity transfer function to obtain the predictive output.

To compare model robustness, five bootstrap data sets under aerobic and anaerobic conditions were applied and compared. Tables 7 and 8 show the means and standard deviations of each variable for the five bootstrap data sets containing 30, 60, 90, 120 and 150 samples. Tables 9 and 10 show that, in all five bootstrap data sets, characteristics represented by mean values of four inputs and one output did not significantly differ from those in the original data set (analysis of variance (ANOVA) test using SPSS 14.0, P>0.05) under aerobic and anaerobic conditions, respectively, which confirmed the reliability of the data selection. Therefore, the 30-bootstrap data set was selected to construct the ANN model and the ANFIS model with 10-fold cross-validation. Figure 9 shows the three-layer feedforward ANN used for comparison. Both the ANN model and the ANFIS model had a four-input (temperature, pH, NaCl, and NaNO_2_) node layer. A weighted logistic transfer function was used to transfer these inputs to a three-node hidden layer. A separate weighted identity transfer function was then used to transfer data from the hidden layer to the one-node (growth rate) output layer. The ANN model was constructed with STATISTICA 10.0 software (StatSoft, Tulsa, OK). The prediction results are further analyzed and discussed below.

**Table 9:** Comparison of original data set and five bootstrap data sets in terms of mean values for four inputs and one output under aerobic conditions (ANOVA test).

Variables	30-bootstrap data set	60-bootstrap data set	90-bootstrap data set	120-bootstrap data set	150-bootstrap data set
	P value	P value	P value	P value	P value
Temperature	0.693	0.684	0.723	0.659	0.590
pH	0.519	0.624	0.563	0.571	0.643
NaCl	0.689	0.971	0.979	0.847	0.836
NaNO_2_	0.934	0.862	0.718	0.838	0.871
Growth rate	0.421	0.542	0.681	0.692	0.617

**Table 10:** Comparison of original data set and five bootstrap data sets in terms of mean values for four inputs and one output under anaerobic conditions (ANOVA test).

Variables	30-bootsrap data set	60-bootstrap data set	90-bootsrap data set	120-bootstrap data set	150-bootstrap data set
	P value	P value	P value	P value	P value
Temperature	0.886	0.902	0.852	0.990	0.879
pH	0.821	0.839	0.977	0.993	0.980
NaCl	0.724	0.926	0.920	0.994	0.965
NaNO_2_	0.465	0.817	0.819	0.627	0.677
Growth rate	0.819	0.852	0.890	0.766	0.853

Tables 11 and 12 show the prediction results. Under aerobic conditions, the averages and standard deviations of the MAPEs were 0.347 and 0.117 in the ANFIS models and 11.041 and 2.576 in the ANN models, respectively. Under anaerobic conditions, the averages and standard deviations of the MAPEs were 0.382 and 0.216 in the ANFIS models and 12.325 and 1.925 in the ANN models, respectively. Under aerobic conditions, the averages and standard deviations of the RMSEs were 0.0016 and 0.0007 in the ANFIS models and 0.035 and 0.008 in the ANN models, respectively. Under anaerobic conditions, the averages and standard deviations of the RMSEs were 0.0016 and 0.0009 in the ANFIS models and 0.036 and 0.006 in the ANN models, respectively. Under aerobic conditions, the averages and standard deviations of the SEPs were 0.564 and 0.214 in the ANFIS models and 0.889 and 0.026 in the ANN models, respectively. Under anaerobic conditions, the averages and standard deviations of the SEPs were 0.569 and 0.315 in the ANFIS models and 14.935 and 2.821 in the ANN models, respectively.

**Table 11:** Comparison of performance indices between ANN and ANFIS models using 30-bootstrap data set with 10-fold cross-validation under aerobic conditions.

Statistical index	Model	Average	Standard deviation	95% C.I.
Mean absolute percentage error (%) (MAPE)	ANN	11.041	2.576	12.983–9.099
	ANFIS	0.347	0.117	0.435–0.259
Root mean square error (RMSE)	ANN	0.035	0.008	0.041–0.029
	ANFIS	0.0016	0.0007	0.0021–0.0011
Standard error of prediction percentage (%) (SEP)	ANN	13.948	3.605	16.666–11.23
	ANFIS	0.564	0.214	0.725–0.403
Bias factor (B_f_)	ANN	0.889	0.026	0.909–0.869
	ANFIS	0.99960	0.00182	1.00097–0.99823
Accuracy factor (A_f_)	ANN	1.126	0.033	1.151–1.101
	ANFIS	1.00348	0.00118	1.00437–1.00259
Absolute fraction of variance (*R* ^2^)	ANN	0.978	0.010	0.986–0.97
	ANFIS	0.99996	0.00003	0.99998–0.99994

**Table 12:** Comparison of performance indices between ANN and ANFIS models using 30-bootstrap data set with 10-fold cross-validation under anaerobic conditions.

Statistical index	Model	Average	Standard deviation	95% C.I.
Mean absolute percentage error (%) (MAPE)	ANN	12.325	1.925	13.776–10.874
	ANFIS	0.382	0.216	0.545–0.219
Root mean square error (RMSE)	ANN	0.036	0.006	0.041–0.031
	ANFIS	0.0016	0.0009	0.0023–0.0009
Standard error of prediction percentage (%) (SEP)	ANN	14.935	2.821	17.062–12.808
	ANFIS	0.569	0.315	0.807–0.331
Bias factor (B_f_)	ANN	0.876	0.019	0.891–0.861
	ANFIS	0.99962	0.00071	1.00015–0.99909
Accuracy factor (A_f_)	ANN	1.142	0.025	1.161–1.123
	ANFIS	1.00383	0.00218	1.00547–1.00219
Absolute fraction of variance (*R* ^2^)	ANN	0.976	0.008	0.982–0.970
	ANFIS	0.99995	0.00005	0.99999–0.99991

The performance comparisons confirmed that the ANFIS models were superior in terms of MAPE, RMSE and SEP (Tables 11 and 12), considering the biological variability associated with the experiment. That is, the results shown in Tables 11 and 12 are consistent with those shown in Tables 5 and 6, respectively, which again confirms the superior accuracy of the ANFIS for predicting *LM* growth rates under aerobic and anaerobic conditions.

**Figure 10:**
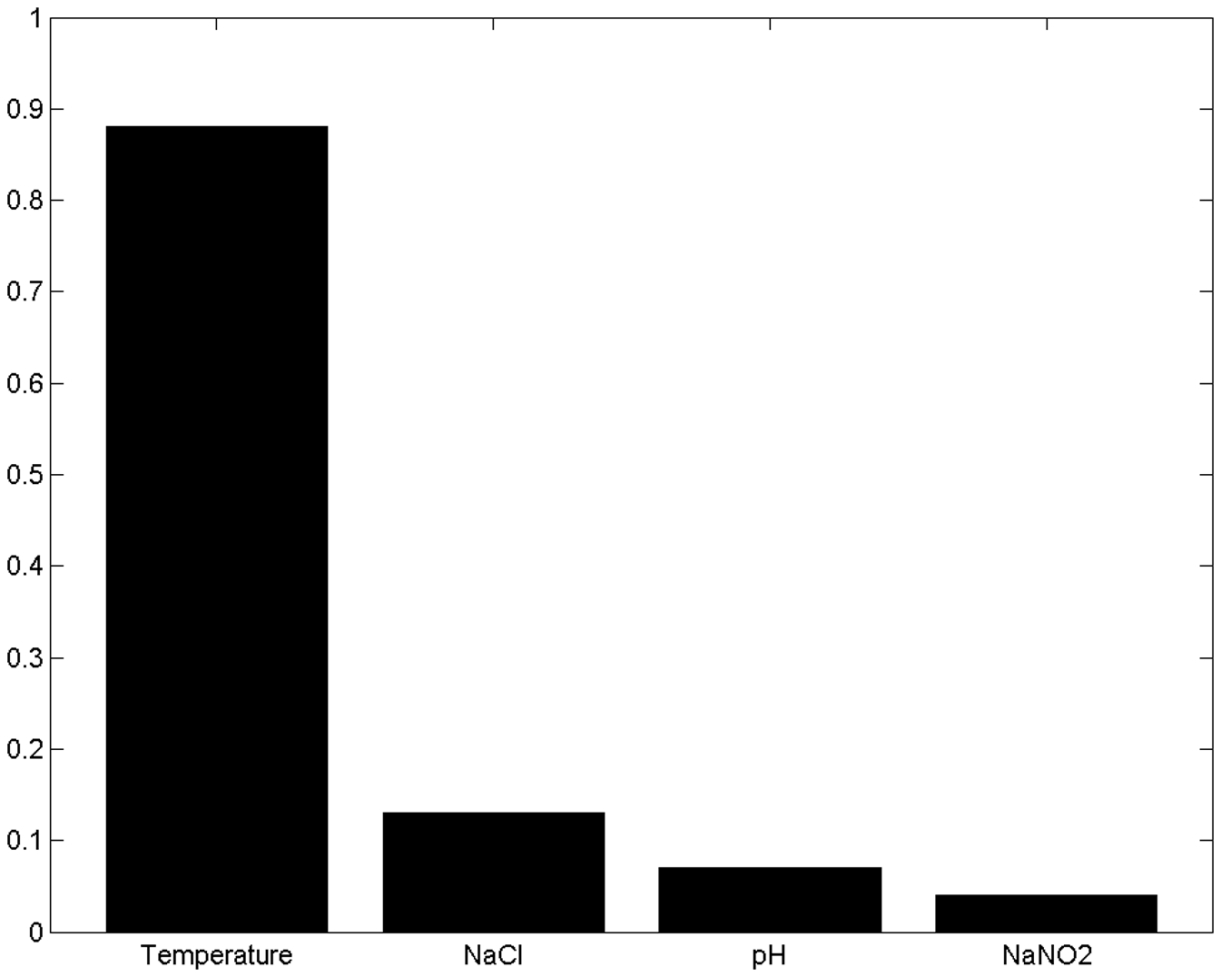
Sensitivity analysis of four input variables under aerobic conditions using ANFIS model. Under aerobic conditions, the sensitivity values for temperature, NaCl, pH and NaCO_2_ were 0.88, 0.13, 0.07 and 0.04, respectively. The most influential (sensitive) parameter affecting the growth rate of *Leuconostoc mesenteroides* under aerobic conditions was temperature and, to a lesser extent, NaCl.

**Figure 11:**
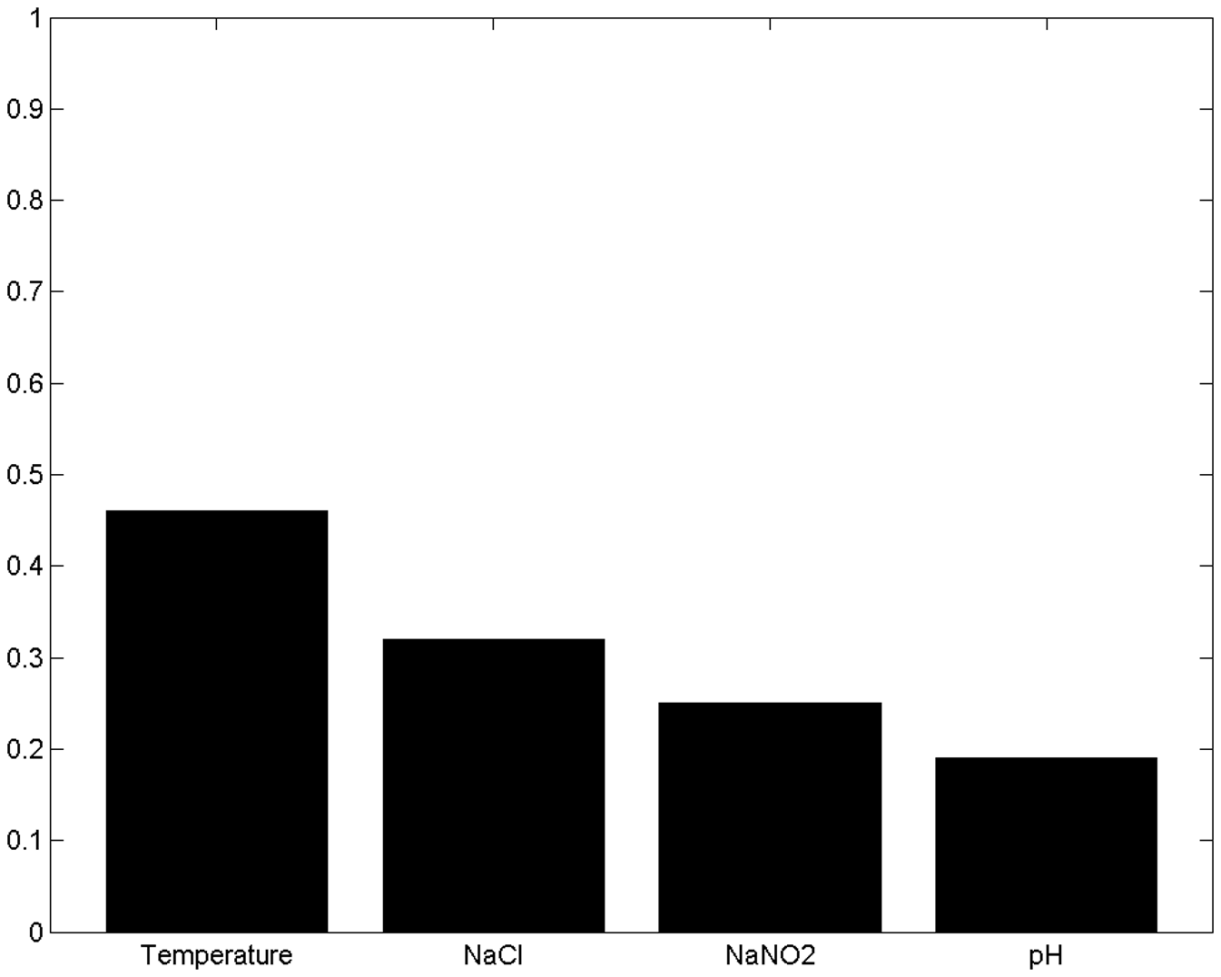
Sensitivity analysis of four input variables under anaerobic conditions using ANFIS model. Under anaerobic conditions, the sensitivity values for temperature, NaCl, NaCO_2_ and pH were 0.46, 0.32, 0.25 and 0.19. The most influential (sensitive) parameters affecting the growth rate of *Leuconostoc mesenteroides* under anaerobic conditions were temperature and, to a lesser extent, NaCl.

Figures 10, 11 12 and 13 show the results of sensitivity analyses of 30-boothstrap data set under both aerobic and anaerobic conditions. In the ANFIS model, the sensitivity values for temperature, NaCl, pH and NaCO_2_ were 0.88, 0.13, 0.07 and 0.04 under aerobic conditions and 0.46, 0.32, 0.19 and 0.25 under anaerobic conditions, respectively. In the ANN model, the sensitivity values for temperature, NaCl, pH and NaCO_2_ were 0.2, 0.06, 0.04 and 0.02 under aerobic conditions and 0.52, 0.16, 0.09 and 0.16 under anaerobic conditions, respectively. That is, the ANFIS and ANN models showed similar results in sensitivity analyses. In terms of effect on the growth rate of *LM* under aerobic and anaerobic conditions, the most influential (sensitive) parameters in both the training and testing data sets were temperature and, to a lesser extent, NaCl.

**Figure 12:**
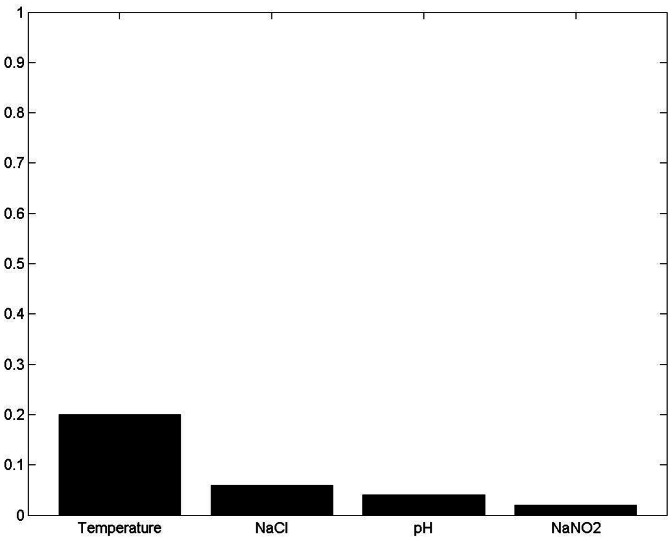
Sensitivity analysis of four input variables under aerobic conditions using ANN model. Under aerobic conditions, the sensitivity values for temperature, NaCl, pH and NaCO_2_ were 0.20, 0.06, 0.04 and 0.02, respectively. The most influential (sensitive) parameter affecting the growth rate of *Leuconostoc mesenteroides* under aerobic conditions was temperature and, to a lesser extent, NaCl.

**Figure 13:**
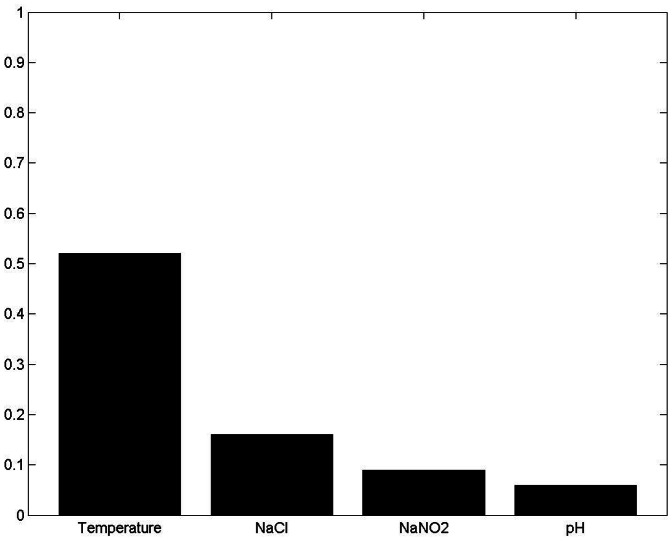
Sensitivity analysis of four input variables under anaerobic conditions using ANN model. Under anaerobic conditions, the sensitivity values for temperature, NaCl, NaCO_2_ and pH were 0.52, 0.16, 0.09 and 0.16. The most influential (sensitive) parameters affecting the growth rate of *Leuconostoc mesenteroides* under anaerobic conditions were temperature and, to a lesser extent, NaCl.

## Discussion

Tables 5 and 6 compare the statistical data for the two models with the original data set, and Figures 5, 6 7 and 8 compare graphical plots of the data. Compared to the ANN model, the ANFIS model showed better agreement with the experimental observations in both the training and testing data sets. The better data fit obtained by the ANFIS-based model in the comparisons with experimental data was confirmed by its *R* ^2^ value of 1.0000 (versus 0.9539–0.9937 in the ANN model) under aerobic conditions and by its *R* ^2^ values of 0.9999–1.0000 (versus 0.9686–0.9989 in the ANN model) under anaerobic conditions.

Notably, although *R* ^2^ is a common criterion for comparing statistical models [32], it assumes a normally distributed error that is independent of the mean value. However, since error distributions are unknown when predicting microbial growth, this term must be used cautiously, particularly in nonlinear regression models [33]. Hence, RMSE values were used for further comparisons of model performance. Under aerobic conditions, the ANFIS model showed better RMSE values (0.001 for the training data set and 0.002 for the testing data set) compared to the ANN model (0.019 for training data set and 0.067 for the testing data set) (Table 5). Under anaerobic conditions, the ANFIS model also showed better RMSE values (0.002 for the training data set and 0.001 for the testing data set) compared to the ANN model (0.008 for training data set and 0.053 for the testing data set) (Table 6). The RMSE, which is calculated by comparing desired and actual output values, which are then averaged across all data, index provides an estimate of goodness of fit in statistical models and indicates the long-term consistency of a model [33]. The lower RMSE values in the ANFIS model in comparison with the ANN model confirmed its superior accuracy in predictions made without previous training.

Like A_f_, MAPE indicates the average deviation from the observed value [26]. In this study, the MAPE results were in good agreement with the A_f_ values estimated for all data sets. In the training data set under aerobic conditions, for instance, the A_f_ values obtained by the ANN and ANFIS models were 1.03 and 1.00, respectively (Table 5), and their average deviations in predicted and actual growth rates were 3% and 0%, respectively. These values were highly consistent with the MAPE values of 3.06% and 0.18% obtained by the ANN and ANFIS models, respectively, in the training data set. Under aerobic conditions, the ANN and ANFIS models obtained A_f_ values of 1.17 and 1.00, respectively (Table 5), and their average deviations in predicted and actual growth rates were 17% and 0%, respectively. Again, these values were closely approximated the MAPE values of 15.69% and 0.27% obtained by the ANN and ANFIS models, respectively, in the testing data set. The relevant figures again confirmed the better performance of the ANFIS models.

The accuracy factor is similar to the B_f_ statistic, which was also introduced by Ross [26]. In this case, a B_f_ value greater than 1 indicates that the model overestimates growth rate and is thus a ‘fail-dangerous’ model whereas a value less than 1 indicates that the model underestimates growth rate and is thus a ‘fail-safe’ model. For example, the accuracy of the growth rate estimates was confirmed by B_f_ values of 1.00 and 1.00 under aerobic and anaerobic conditions, respectively, in the ANFIS model and by B_f_ values of 0.99 and 1.00 under aerobic and anaerobic conditions, respectively, in the ANN models (Tables 5 and 6). These data indicate that the ANFIS model provides a relatively more accurate estimate of growth rate compared to the ANN model, which tends to underestimate growth rate.

The SEP index indicates the relative deviation in mean prediction values. A notable advantage of the index is that its calculation is independent of the magnitude of the measurements [10]. For both training and testing data sets (Tables 5 and 6), the SEP indices were again better in the ANFIS models (range, 0.44–0.81) than in the ANN models (range, 3.66–23.27).

Tables 11 and 12 show the evaluation criteria for ANFIS and ANN models with 10-fold cross-validation of the 30-bootstrap data set. The tables also show that the ANFIS models were better than the ANN models in predicting the four kinetic parameters.

The ANFIS and ANN models offer an interesting option for defining the sensitivity and relative importance of inputs (Figures 10, 11, 12 and 13). The sensitivity analysis in this experiment revealed the important effects of temperature on *LM* growth rates under aerobic and anaerobic conditions. In earlier works, Zurera-Cosano et al. [9] obtained similar results in RSM models of *LM* growth whereas Panagou and Kodogiannis [7] reported similar results in an ANN model of *Monascus ruber* growth.

In conclusion, this study confirmed that, compared to ANN models, ANFIS architectures provide better accuracy in predicting the growth rate of *LM* based on input data for temperature, pH, NaCl and NaNO_2_ under both aerobic and anaerobic conditions. The statistical indices and graphic plots confirmed the superior performance of the ANFIS models in both training and testing data sets. Sensitivity analyses of the ANFIS models revealed that the most influential (sensitive) factors in the growth rate of *LM* were temperature and, to a lesser extent, NaCl. As ANFIS is mainly applied in predictive microbiology, the findings that ANFIS models are effective for predicting the kinetic parameters of fungi indicate their good potential use as an alternative to ANNs in this field. Future studies of differential evolution [34] in the ANFIS architecture may evaluate the effects of training on nonlinear parameters of the premise part and on linear parameters of the consequent part. Hopefully, improving the accuracy of the model will enhance its potential applications in predictive microbiology.

## References

[1] Ross T, McMeekin TA (1994) Review paper: predictive microbiology. International Journal of Food Microbiology 23: 241–264. 10.1016/0168-1605(94)90155-4 7873329

[2] Gibson AM, Hocking AD (1997) Advances in the predictive modelling of fungal growth in food. Trends in Food Science and Technology 8: 353–358.

[3] Murphy PA, Hendrich S, Landgren C, Bryant CM (2006) Food mycotoxins: an update. Journal of Food Science 71: 51–65.

[4] Dantigny P, Guilmart A, Bensoussan M (2005) Basis of predictive mycology. International Journal of Food Microbiology 100: 187–196. 15854704 10.1016/j.ijfoodmicro.2004.10.013

[5] Parra R, Magan N (2004) Modelling the effect of temperature and water activity on growth of *Aspergillus niger* strains and applications for food spoilage moulds. Journal of Applied Microbiology 97: 429–438. 15239711 10.1111/j.1365-2672.2004.02320.x

[6] Patriarca A, Vaamonde G, Fernandez Pinto V, Comerio R (2001) Influence of water activity and temperature on the growth of Wallemia sebi: application of a predictive model. International Journal of Food Microbiology 68: 61–67. 11545221 10.1016/s0168-1605(01)00470-6

[7] Panagou EZ, Kodogiannis VS (2009) Application of neural networks as a non-linear modelling technique in food mycology. Expert Systems with Applications 36: 121–131.

[8] Huis in't Veld JHJ (1996) Microbial and biochemical spoilage of foods: an overview. International Journal of Food Microbiology 33: 1–18. 8913806 10.1016/0168-1605(96)01139-7

[9] Zurera-Cosano G, Garcia-Gimeno RM, Rodriguez-Perez R, Hervas-Martinez C (2006) Performance of response surface model for prediction of *Leuconostoc mesenteroides* growth parameters under different experimental conditions. Food Control 17: 429–438.

[10] Garcia-Gimeno RM, Hervas-Martinez C, Rodriguez-Perez R, Zurera-Cosano G (2005) Modelling the growth of *Leuconostoc mesenteroides* by artificial neural networks. International Journal of Food Microbiology 105: 317–332. 16054719 10.1016/j.ijfoodmicro.2005.04.013

[11] Tsai JT, Chou JH, Liu TK (2006) Tuning the structure and parameters of a neural network by using hybrid Taguchi-genetic algorithm. IEEE Trans on Neural Networks 17: 69–80. 16526477 10.1109/TNN.2005.860885

[12] Ho WH, Tsai JT, Hsu GM, Chou JH (2010) Process parameters optimization: a design study for TiO_2_ thin film of vacuum sputtering process. IEEE Trans on Automation Science and Engineering 7: 143–146.

[13] Ho WH, Chang CS (2011) Genetic-algorithm-based artificial neural network modeling for platelet transfusion requirements on acute myeloblastic leukemia patients. Expert Systems With Applications 38: 6319–6323.

[14] Ho WH, Lee KT, Chen HY, Ho TW, Chiu HC (2012) Disease-free survival after hepatic resection in hepatocellular carcinoma patients: a prediction approach using artificial neural network. PLoS ONE 7: e29179. 22235270 10.1371/journal.pone.0029179 PMC3250424

[15] Shi HY, KT Lee, HH Lee, WH Ho, DP Sun, et al (2012) Comparison of artificial neural network and logistic regression models for predicting in-hospital mortality after primary liver cancer surgery. PLoS ONE 7: e35781. 22563399 10.1371/journal.pone.0035781 PMC3338531

[16] Hajmeer MN, Basheer IA, Najjar YM (1997) Computational neural networks for predictive microbiology II. Application to microbial growth. International Journal of Food Microbiology 34: 51–66. 9029255 10.1016/s0168-1605(96)01169-5

[17] Geeraerd AH, Herremans CH, Cenens C, Van Impe JF (1998) Application of artificial neural networks as a non-linear modular modelling technique to describe bacterial growth in chilled food products. International Journal of Food Microbiology 44: 49–68. 9849784 10.1016/s0168-1605(98)00127-5

[18] Jeyamkondan S, Jayas DS, Holley RA (2001) Microbial growth modelling with artificial neural networks. International Journal of Food Microbiology 64: 343–354. 11294356 10.1016/s0168-1605(00)00483-9

[19] Lou W, Nakai S (2001) Application of artificial neural networks for predicting the thermal inactivation of bacteria: a combined effect of temperature, pH and water activity. Food Research International 34: 573–579.

[20] Lou W, Nakai S (2001) Artificial neural network-based predictive model for bacterial growth in a simulated medium of modified atmosphere packed cooked meat products. Journal of Agricultural and Food Chemistry 49: 1799–1804. 11308328 10.1021/jf000650m

[21] Garcia-Gimeno RM, Hervas-Martinez C, Barco-Alcala E, Zurera-Cosano G, Sanz-Tapia E (2003) An artificial neural network approach to *Escherichia coli* O157:H7 growth estimation. Journal of Food Science 68: 639–645.

[22] Jang JSR (1993) ANFIS: adaptive network-based fuzzy inference systems. IEEE Transactions on Systems, Man, and Cybernetics 23: 665–685.

[23] Ho WH, Chen JX, Lee IN, Su HC (2011) An ANFIS-based model for predicting adequacy of vancomycin regimen using improved genetic algorithm. Expert Systems With Applications 38: 13050–13056.

[24] Lo SP (2003) An adaptive-network based fuzzy inference system for prediction of workpiece surface roughness in end milling. Journal of Materials Processing Technology 142: 665–675.

[25] Ertunc HM, Hosoz M (2008) Comparative analysis of an evaporative condenser using artificial neural network and adaptive neuro-fuzzy inference system. International Journal of Refrigeration 31: 1426–1436.

[26] Ross T (1996) Indices for performance evaluation of predictive models in food microbiology. Journal of Applied Bacteriology 81: 501–508. 8939028 10.1111/j.1365-2672.1996.tb03539.x

[27] Ala-Korpela M, Hiltunen Y, Bell JD (1995) Quantification of biomedical NMR data using artificial neural network analysis: lipoprotein lipid profiles from 1H NMR data of human plasma. NMR Biomed 8: 235–244. 8732179 10.1002/nbm.1940080603

[28] Piscaglia F, Cucchetti A, Benlloch S, Vivarelli M, Berenguer J, et al (2006) Prediction of significant fibrosis in hepatitis C virus infected liver transplant recipients by artificial neural network analysis of clinical factors. European Journal of Gastroenterology and Hepatology 18: 1255–1261. 17099373 10.1097/01.meg.0000243885.55562.7e

[29] Efron B, Tibshirani R (1993) An introduction to the bootstrap. New York, USA: Chapman and Hall.

[30] Akins RB, Tolson H, Cole BR (2005) Stability of response characteristics of a Delphi panel: application of bootstrap data expansion. BMC Medical Research Methodology 5: 37. 16321161 10.1186/1471-2288-5-37 PMC1318466

[31] MATLAB Documentation (2002) Fuzzy Toolbox User's Guide of MATLAB. The MathWorks, Inc.

[32] Buchanan RL, Bagi LK (1994) Expansion of response surface models for the growth of *Escherichia coli* O157:H7 to include sodium nitrite as a variable. International Journal of Food Microbiology 23: 317–332. 7873334 10.1016/0168-1605(94)90160-0

[33] Ratkowsky DA (1990) Handbook of nonlinear regression models. New York, USA: Marcel Dekker Inc.

[34] Ho WH, Chou JH, Guo CY (2010) Parameter identification of chaotic systems using improved differential evolution algorithm. Nonlinear Dynamics 61: 29–41.

